# Predicting atrial fibrillation in primary care using machine learning

**DOI:** 10.1371/journal.pone.0224582

**Published:** 2019-11-01

**Authors:** Nathan R. Hill, Daniel Ayoubkhani, Phil McEwan, Daniel M. Sugrue, Usman Farooqui, Steven Lister, Matthew Lumley, Ameet Bakhai, Alexander T. Cohen, Mark O’Neill, David Clifton, Jason Gordon

**Affiliations:** 1 Bristol-Myers Squibb Pharmaceutical Ltd, Uxbridge, United Kingdom; 2 Health Economics and Outcomes Research Ltd, Cardiff, United Kingdom; 3 Pfizer Ltd, Surrey, United Kingdom; 4 Department of Cardiology, Royal Free Hospital, London, United Kingdom; 5 Department of Haematological Medicine, Guys and St Thomas' NHS Foundation Trust, King's College London, London, United Kingdom; 6 Division of Cardiovascular Medicine, Guys and St Thomas' NHS Foundation Trust, King's College London, London, United Kingdom; 7 Department of Engineering Science, University of Oxford, Oxford, United Kingdom; University of Alabama at Birmingham, UNITED STATES

## Abstract

**Background:**

Atrial fibrillation (AF) is the most common sustained heart arrhythmia. However, as many cases are asymptomatic, a large proportion of patients remain undiagnosed until serious complications arise. Efficient, cost-effective detection of the undiagnosed may be supported by risk-prediction models relating patient factors to AF risk. However, there exists a need for an implementable risk model that is contemporaneous and informed by routinely collected patient data, reflecting the real-world pathology of AF.

**Methods:**

This study sought to develop and evaluate novel and conventional statistical and machine learning models for risk-predication of AF. This was a retrospective, cohort study of adults (aged ≥30 years) without a history of AF, listed on the Clinical Practice Research Datalink, from January 2006 to December 2016. Models evaluated included published risk models (Framingham, ARIC, CHARGE-AF), machine learning models, which evaluated baseline and time-updated information (neural network, LASSO, random forests, support vector machines), and Cox regression.

**Results:**

Analysis of 2,994,837 individuals (3.2% AF) identified time-varying neural networks as the optimal model achieving an AUROC of 0.827 vs. 0.725, with number needed to screen of 9 vs. 13 patients at 75% sensitivity, when compared with the best existing model CHARGE-AF. The optimal model confirmed known baseline risk factors (age, previous cardiovascular disease, antihypertensive medication usage) and identified additional time-varying predictors (proximity of cardiovascular events, body mass index (both levels and changes), pulse pressure, and the frequency of blood pressure measurements).

**Conclusion:**

The optimal time-varying machine learning model exhibited greater predictive performance than existing AF risk models and reflected known and new patient risk factors for AF.

## Introduction

Atrial fibrillation (AF), the most common sustained heart arrhythmia[1], is associated with an approximately five-fold increase in stroke[2] and an increase in stroke severity compared to non-AF patients, resulting in higher morbidity and mortality[3, 4]. Prevalence of AF is estimated globally at 46.3 million, with 3.8 million new diagnoses annually[5]. However, true prevalence of the condition is likely to be higher, as paroxysmal, minimally symptomatic and asymptomatic AF can be difficult to diagnose, and consequently AF is often diagnosed incidentally. In England alone, 425,000 people are estimated to be living with undiagnosed AF[6]. Given that these patients are at increased risk of stroke-related death or disability,[3, 4] early detection and effective management of AF have the potential to improve patient outcomes and alleviate the economic burden of AF and its sequelae.

Both European and US guidelines recommend diagnosing AF based on a 12-lead electrocardiogram (ECG) or rhythm strip[7, 8]; however, 12-lead ECG use for AF detection in primary care has been reported not to be cost-effective, regardless of whether used in a systematic (e.g. patients >65 years) or targeted (including high-risk patients only) approach[9]. At present, European guidelines recommend opportunistic identification by pulse check in patients aged >65 years, reserving 12-lead ECG or rhythm strip for confirmatory examination[7]. However, pulse-checking lacks diagnostic precision, so many patients undergo further testing unnecessarily[10]. There is therefore an interest in narrowing the patient population that should undergo detailed testing for AF to ensure maximum detection of AF cases while maintaining cost-effectiveness.

One approach to improve the precision of patient identification is by applying published risk models that use baseline clinical variables and biomarkers for future AF risk prediction[11–13] or computerised tools designed to predict AF based on data routinely collected in the clinic[14]. However, several of these models depend on ECG-derived data[12, 13] and none are automated therefore requiring input by healthcare providers[11–13].

Machine learning is a data-driven approach that can identify nonlinear associations and complex interactions between variables without the need to pre-specify these relationships *a priori*. The use of machine learning for predicting disease may enable physicians to speed-up decision making and improve efficiency, reliability, and accuracy. In line with published applications of machine learning in various aspects of healthcare[15], we utilised machine learning techniques to develop a risk model, that is contemporaneous and informed by routinely collected patient data, and that has the potential to be used as a predictive tool in primary care to identify people at high risk of AF that have not yet been diagnosed.

## Methods

### Study design

This was a retrospective, observational cohort study using primary care data obtained from the UK Clinical Practice Research Datalink (CPRD)[16]. The study population included adults registered at practices included within the CPRD who were ≥30 years and with no history of AF in five-years prior to the study period (January 2006 to December 2016). Diagnoses of AF (or atrial flutter, to account for mis-recording) and other comorbidities were identified using Read codes (S1 Table in the supporting information). All data provided by CPRD was fully deanonymized. The study protocol was reviewed and approved by the Independent Scientific Advisory Committee for MHRA database research (reference 17_151).

The primary aim of this study was to develop a clinically applicable risk prediction model to identify associations between baseline and time-varying factors and the identification of AF. Development and evaluation of the optimum risk model for prediction of AF was a three-stage process. In the first stage of model building, we evaluated different baseline models including logistic least absolute shrinkage and selector operator (LASSO)[17], random forests[18], support vector machines[19], neural networks (NN)[20], Cox regression and published AF risk models (Framingham[13], ARIC[12] and CHARGE-AF[11]). At this stage, index date was defined as time at which patients could be adequately characterised, i.e., when a complete set of key clinical measurements (height, weight, body mass index (BMI), systolic blood pressure [SBP], and diastolic blood pressure [DBP]) was recorded within a rolling 12-month window ending during the study period. Patients were followed up until the earliest of: AF diagnosis; death; loss to follow-up; or end of study (S1 Fig).

Stage 1 confirmed that machine learning techniques identified similar associations to other baseline models evaluated. Given this, the second stage of model building focused upon the use of machine learning to identify time-varying associations not uncovered by the baseline models. The NN was the optimum approach identified at stage 1 (S2 Table) and was taken forward to stage 2 whereby time-varying covariates were considered (S3 Table), to reflect the evolution of AF risk factors prior to an AF diagnosis. A case-control sample was used by sub-setting the stage 1 population to patients with AF during follow-up and an age- and sex-matched control group. The index date for the time-varying model was date of AF diagnosis, or equivalent for matched non-AF controls (S1 Fig).

The third stage of model building combined the two models (baseline and time-varying NN) into a single risk model to create a clinically relevant and applicable risk prediction algorithm.

All analyses were conducted using R v3.3.1[21].

### Candidate covariates

The covariates considered in the models were limited to those included in existing AF risk models[11–13] (S4 Table). In order to facilitate implementation of the resulting model, all covariates were derived from Read codes, thereby utilising data that are routinely collected in clinical practice.

Covariates in the Cox models and logistic models were selected using p-values of the estimated coefficients, with p<0.05 as the selection criterion. Covariates in the machine learning approaches at both stages 1 and 2 were selected by recursive feature elimination, with hyper-parameters being optimised through five-fold cross-validation (S5 Table)[22].

For the baseline model, covariates were taken to be the latest value recorded in the one year (365.25 days) prior to study entry. For the time-varying model, extracted data was structured in 91-day quarters working backwards over a one-year period from AF diagnosis (index) date to reflect evolution of risk factors in the year prior to AF diagnosis (three years of data were considered, but no substantial increase in performance was observed when using data beyond the first year). Last-observation-carried-forward was used to impute clinical measurements in quarters with no data.

### Assessment of model performance

The performance of the final risk model (stage 3) was evaluated on the stage 2 population, to reflect the evolution of the risk factors in the model’s predictions. Inverse-probability weights were applied to ensure the resulting performance metrics were representative of the full study population observed at stage 1, rather than the artificially balanced case-control sample at stage 2. The weights were equal to 1 for patients with AF over follow-up (as all these patients were included in the sample), and calculated as the inverse selection probability for patients without AF, ensuring the original age and sex distributions were recovered.

To aid generalizability of the results beyond the sample to which the risk models were fitted, patients were randomly assigned to training and holdout datasets in a 2:1 ratio; these datasets were used to fit and independently assess each risk model, respectively.

To aid interpretability of the fitted NN, each covariate’s “relative strength” in predicting AF risk was quantified[23] and expressed as a percentage of the most important covariate within the model. Partial dependence plots[24] were used to assess the size and direction of each covariate’s association with AF incidence, whereby AF probabilities were generated by varying each covariate of interest and holding all other covariates fixed at their observed patient-level values.

Discrimination between AF and non-AF cases was assessed using area under the receiver operating characteristic curve (AUROC): value in the range 0–1, where 1 indicates perfect separation of AF and non-AF cases. Positive predictive value (PPV) and potential number-needed-to-screen (NNS)[25] (potential due to the diagnostic accuracy of the arrhythmia detection device and the paroxysmal nature of the disease) were computed at sensitivities of 25%, 50%, and 75%. Sensitivity analyses excluded time-varying comorbidities (S4 Table) as candidate predictors of AF diagnosis.

The performance of the optimum NN model was evaluated by comparison with the current best-performing of the pre-existing models to predict risk at baseline namely the CHARGE-AF risk equation, which uses classical statistical methods.

## Results

### Study population patient characteristics

A total of 2,994,837 patients were included in the baseline model (S2 Fig), with 162,672 included in the time-varying model (AF patients >1 year of follow-up and matched non-AF sample). Over the 11-year study period, 95,607 patients (3.19%) were diagnosed with AF, median follow-up time was 7.09 years, and a total of 19.63 million patient-years were accrued.

Mean age at baseline for patients diagnosed with AF was 70.2 years, compared to 55.5 years for non-AF (Table 1). AF patients were more likely to be male and former smokers than non-AF patients, and a greater proportion of AF patients had a history of each evaluated comorbidity at baseline compared to non-AF patients. AF patients had a mean baseline BMI and SBP of 28.6 kg/m^2^ and 141.0 mmHg, respectively, compared to 27.6 kg/m^2^ and 133.3 mmHg for non-AF patients. There was no clinically meaningful difference in mean baseline DBP between AF and non-AF patients (79.1 mmHg and 79.4 mmHg, respectively), but mean pulse pressure was notably greater for AF patients, at 61.9 mmHg compared to 53.9 mmHg for non-AF patients. Differences in baseline characteristics between AF and non-AF patients were statistically significant for all variables considered, reflecting the large analysed sample. However, only age was observed to have a ‘large’ effect size (Cohen’s *d* ≥ 0.8) according to Cohen (1988)[26], while only history of hypertension met the d ≥ 0.5 threshold defined by Cohen (1988)[26] as being indicative of a ‘medium’ effect size.

**Table 1:** Patient disposition at study entry.

Variable	Category	Full population (n = 2,994,837)	AF cohort (n = 95,607)	Non-AF cohort (n = 2,899,230)	P-value	Cohen’s *d*
**Demographic characteristics**						
Age (years), mean (SD)		55.98 (14.46)	70.23 (11.07)	55.51 (14.32)	<0.001	1.0343
Sex, n (%)	Male	1,395,397 (46.6)	51,738 (54.1)	1,343,659 (46.3)	<0.001	0.1558
Smoking status, n (%)	Current	555,074 (18.5)	10,571 (11.1)	544,503 (18.8)	<0.001	-0.1989
	Former	701,966 (23.4)	32,198 (33.7)	669,768 (23.1)		0.2499
	Non-smoker	1,269,538 (42.4)	37,384 (39.1)	1,232,154 (42.5)		-0.0688
	Passive	7,876 (0.3)	279 (0.3)	7,597 (0.3)		0.0058
	Unknown	460,383 (15.4)	15,175 (15.9)	445,208 (15.4)		0.0143
Race, n (%)	Black	29,380 (1.0)	207 (0.2)	29,173 (1.0)	<0.001	-0.0801
	White	729,507 (24.4)	25,459 (26.6)	704,048 (24.3)		0.0546
	Other	75,142 (2.5)	1,031 (1.1)	74,111 (2.6)		-0.0945
	Unknown	2,160,808 (72.2)	68,910 (72.1)	2,091,898 (72.2)		-0.0017
Clinical histories*, n (%)						
Hypertension		748,849 (25.0)	50,501 (52.8)	698,348 (24.1)	<0.001	0.6306
Heart failure		22,054 (0.7)	2,805 (2.9)	19,249 (0.7)	<0.001	0.2658
Left ventricular hypertrophy		4,727 (0.2)	502 (0.5)	4,225 (0.1)	<0.001	0.0956
Myocardial infarction		42,830 (1.4)	3,009 (3.1)	39,821 (1.4)	<0.001	0.1494
Coronary heart disease		154,029 (5.1)	13,703 (14.3)	140,326 (4.8)	<0.001	0.4310
Congenital heart disease		501 (<0.1)	58 (0.1)	443 (<0.1)	<0.001	0.0351
Type 1 diabetes		19,101 (0.6)	831 (0.9)	18,270 (0.6)	<0.001	0.0300
Type 2 diabetes		187,733 (6.3)	10,727 (11.2)	177,006 (6.1)	<0.001	0.2111
**Clinical measurements, mean (SD)**						
Height (m)		1.68 (0.10)	1.69 (0.11)	1.68 (0.10)	<0.001	0.0508
Weight (kg)		78.32 (18.28)	81.55 (19.45)	78.21 (18.23)	<0.001	0.1825
BMI (kg/m^2^)		27.59 (5.99)	28.56 (6.16)	27.56 (5.99)	<0.001	0.1662
SBP (mmHg)		133.58 (18.87)	140.97 (19.29)	133.34 (18.81)	<0.001	0.4054
DBP (mmHg)		79.40 (10.92)	79.12 (11.01)	79.41 (10.92)	<0.001	-0.0266
Pulse pressure (mmHg)		54.18 (14.93)	61.85 (16.47)	53.93 (14.81)	<0.001	0.4917

AF: atrial fibrillation and atrial flutter; BMI: body mass index; DBP: diastolic blood pressure; SBP: systolic blood pressure; SD: standard deviation.

*clinical histories assessed five years prior to index.

### Inferences from machine learning models

**Fig 1:**
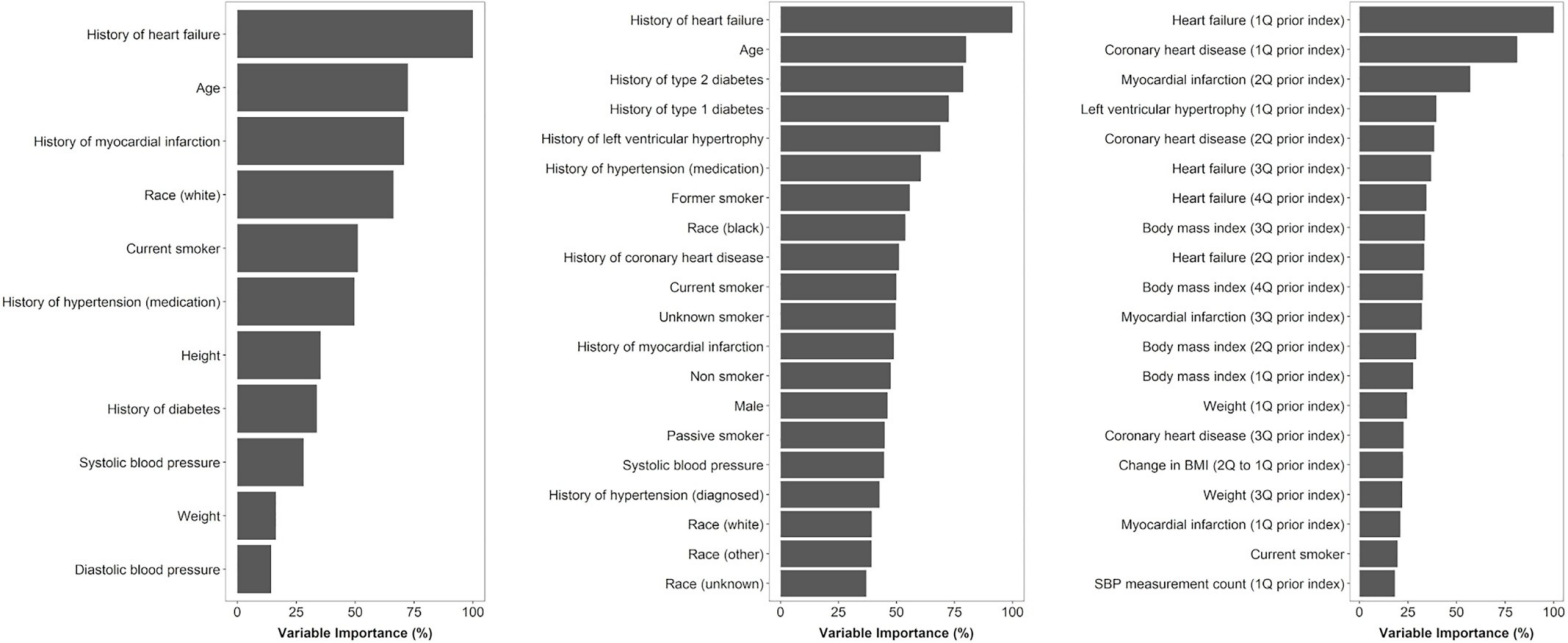
**Most important predictors of AF according to: (A) the CHARGE-AF risk model; (B) the fitted baseline neural network; and (C) the fitted time-varying neural network.** Variable importance was determined on the training dataset according to the absolute size of the published regression coefficients for the CHARGE-AF risk model[11] and Garson’s algorithm[23] for the fitted neural networks. Importance is expressed as a percentage of the most important predictor within each model. Importance is shown for all 11 variables in the CHARGE-AF risk model and the top 20 most important variables in each of the fitted neural network models.

A total of 24 and 100 predictors of AF were used in the fitted baseline (stage 1) and time-varying (stage 2) NNs, respectively; key risk factors elicited from these models are summarised in Table 2. When ranked by importance, top predictors in the baseline model were age, history of cardiovascular diseases and events, and prescriptions for antihypertensive medications (Fig 1).

**Table 2:** Summary of key AF risk factors inferred from the final risk model.

Risk factor
Patient demographics (age, sex, race, smoking status) at baseline
History of antihypertensive medication use at baseline
History of type 1 or type 2 diabetes at baseline
History of cardiovascular comorbidities at baseline
Presence of a cardiovascular event in the past year
BMI in each of the latest four quarters
Change in BMI between the latest two quarters
High pulse pressure in the latest quarter
Negative absolute change in DBP or positive absolute change in SBP between the latest two quarters
Increasing frequency of DBP, SBP and BMI recording in the latest quarter

BMI: body mass index; DBP: diastolic blood pressure; SBP: systolic blood pressure. Risk factors were identified using model inferences from variable importance plots[23] and partial dependence plots[24] for the fitted baseline and time-varying neural networks. Smoking status was defined as: current smoker, former smoker, non-smoker, passive smoker and unknown. Cardiovascular comorbidities/events considered were: hypertension (diagnosed), hypertension (receiving antihypertensive medication), heart failure, coronary heart disease, congenital heart disease, myocardial infarction left ventricular hypertrophy, type 1 diabetes, type 2 diabetes. Pulse pressure is the difference between systolic and diastolic blood pressure, corresponding to the force generated by cardiac contraction. “High” pulse pressure refers to elevated (>120 mmHg) SBP combined with a low to normal (≤80 mmHg) DBP. For details, see S3 Fig. Frequency with which clinical characteristics were recorded was a continuous variable representing the number (count) of DBP, SBP, etc. recordings, rather than actual values recorded over a given quarter.

In the time-varying NN, the presence of heart failure in the most recent 91-day quarter contributed most to the prediction of AF diagnoses (Fig 1); other adverse cardiovascular events (coronary heart disease, myocardial infarction, and left ventricular hypertrophy) in the past year were also important predictors. Values of BMI observed across the previous four quarters were important predictors of AF, as was the change in BMI between the two most recent quarters. The number of SBP measurements recorded in the latest quarter made a greater contribution to the model than the SBP value itself, and the frequency of recording was non-linearly associated with AF diagnosis, with similar association patterns also being observed for the frequency of DBP and BMI recording in the latest quarter (S3 Fig). A substantial fall in DBP or an increase in SBP, which is indicative of increased pulse pressure, between the two latest quarters was associated with AF diagnosis, as was having high SBP and low-normal DBP in the most recent quarter (S3 Fig).

### Performance

**Fig 2:**
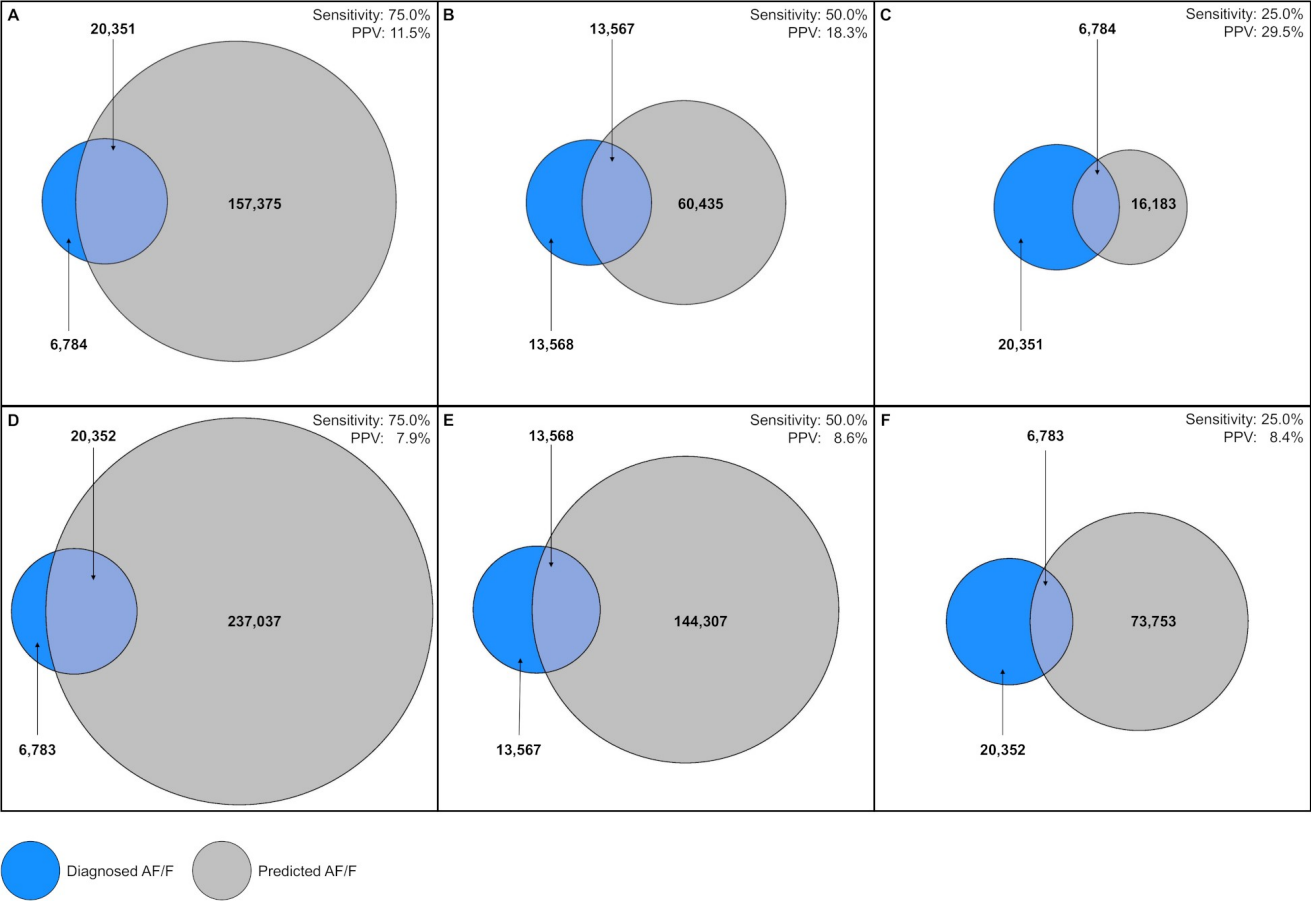
Illustration of the trade-off between sensitivity and positive predictive value (PPV) for the final machine learning risk model (A–C) compared with the baseline CHARGE-AF risk model (D–F).

To identify 75% of diagnosed AF cases, the final NN (incorporating predicted probabilities from the baseline and time-varying NN) achieved a PPV of 11.5%, compared to 7.9% for CHARGE-AF and 6.5% for logistic regression (Table 3), corresponding to NNS of 9, 13, and 15 patients, respectively. Improved precision of the NN over CHARGE-AF was also observed at sensitivity thresholds of 25% and 50% (Fig 2).

**Table 3:** Assessment of model performance at 75% sensitivity.

Model	Specificity *(Relative change vs*. *LR)*	PPV *(Relative change vs*. *LR)*	NNS *(Change* *vs*. *LR)*	AUROC *(Relative change vs*. *LR)*
Final machine learning risk model	74.9% *(+44*.*0%)*	11.5% *(+76*.*9%)*	9 patients *(-6 patients)*	0.827 *(+19*.*0%)*
CHARGE-AF risk model	61.0% *(+17*.*3%)*	7.9% *(+21*.*5%)*	13 patients *(-2 patients)*	0.725 *(+4*.*3%)*
Logistic regression	52.0%	6.5%	15 patients	0.695

AUROC: area under the receiver operating characteristic curve; LR: logistic regression; NNS: number needed to screen (number of patients needed to be screened to identify one AF case); PPV: positive predictive value (percentage of screened patients diagnosed with AF). Sensitivity: percentage of patients diagnosed with AF that would be identified for screening; specificity: percentage of patients not diagnosed with AF that would not be identified for screening.

The blue circles represent the 27,135 patients diagnosed with AF, while the grey circles represent patients flagged as being at risk of AF by each of the models. The overlap between the blue and grey circles represents a given level of sensitivity (the percentage of diagnosed AF cases that are detected by the model, fixed at 75%, 50% or 25%); the resulting PPV (the percentage of patients flagged by the model that are diagnosed with AF) is represented by the proportion of the grey circle that lies within the overlapping region. The figure therefore demonstrates that, for a given level of sensitivity, our risk model achieves a greater PPV than CHARGE-AF, implying that fewer patients would need to be screened to identify the same number of AF cases. For example, to identify 75% of patients with AF (20,351 true-positives / [20,351 true-positives + 6,784 false-negatives]), our risk model would flag 177,726 patients (157,375 false-positives + 20,351 true-positives) to be screened (panel A). In comparison, to identify the same proportion of AF cases, CHARGE-AF would flag 257,389 patients (237,037 false-positives + 20,352 true-positives) to be screened (panel D).

The patient numbers displayed relate to the stage 2 holdout dataset after applying post-stratification weights to reflect the AF, age and sex composition of the full eligible population. A total of 627,768 patients were included in the analysis after weighting; the difference between this total and the sum of the values shown in the figure constitutes true negative cases (patients not flagged and not diagnosed with AF).

The NN achieved an AUROC of 0.827, compared to 0.725 for CHARGE-AF and 0.695 for logistic regression (Table 3). When time-varying cardiovascular comorbidities were excluded as candidate predictors of AF, the resulting AUROC of the time-varying model fell to 0.812, a relative decrease of just 0.7%, indicating that the remaining time-varying covariates are themselves strong predictors of AF risk.

## Discussion

The developed risk model has a high yield and potential to reduce greatly the number of patients being screened for AF. When compared with the best performing existing model, CHARGE-AF, our risk model reduced the potential NNS by 31% from 13 to 9, equating to 79,663 fewer patients in total. The model had an AUROC of 0.827, a 14.1% relative increase in its ability to discriminate between diagnosed AF and non-AF cases when compared with an AUROC 0.725 for CHARGE-AF. Notably, the relative increase in AUROC achieved by NN over traditional approaches when only baseline covariates were considered was far more modest (2.8% versus CHARGE-AF and 2.6% versus Cox regression), indicative of the potential value of combining machine learning with time-varying covariates. However, even a minor improvement will have tangible implications for patient outcomes in clinical practice, either through unnecessarily screening fewer patients or capturing more cases of AF. The final risk model provided a superior trade-off between sensitivity and precision compared with all the existing AF risk models, and could therefore refine target populations that warrant further investigations to diagnose AF. These results add to the growing body of evidence supporting the use of machine learning techniques across various therapeutic areas[15].

Rather than mining the CPRD for new AF risk factors, possibly without clinical rationale, candidate predictors considered in our machine learning models were derived from three existing risk models (CHARGE-AF[11], Framingham[13] and ARIC[12]). The risk factors emerging from our baseline model are therefore already well established, and include patient demographics[11–13], heart failure[11–13], diabetes[11, 12], left ventricular hypertrophy[11, 12], coronary heart disease[12], myocardial infarction[11], antihypertensive treatment[11–13], and SBP[11–13]. A history of smoking was more strongly associated with AF risk than current smoking, possibly reflecting patients with poorer health preferentially giving up smoking. Similarly, antihypertensive treatment prescription was more influential than a diagnosis of hypertension (which may be affected by varying hypertension definitions) or measured SBP (influenced by short-term intra-patient variability). These factors may also be associated with more frequent medical contact or pulse characterisation inputs and greater opportunities for AF diagnosis.

Classical statistical methods, as were applied in the CHARGE-AF[11] risk model, assume linear relationships between covariates and predicted risk, and interactions between covariates must be explicitly pre-specified. An advantage of machine learning is its ability to identify highly non-linear associations between covariates and incidence, and empirically detect interactions between variables from observed data. For example, we observed a non-linear association between the frequency of SBP recording in the most recent quarter and risk of AF. Interestingly, the number of records in the most recent quarter appeared more important than the absolute measure. This may also be due to patients with comorbidities or underlying ill health having more frequent contact with the healthcare system, especially prior to disease diagnosis. There was an interaction between the most recently recorded DBP and SBP values, whereby high SBP coupled with low-normal DBP was associated with increased risk of AF. This supports the previously described notion that high pulse pressure is a risk factor for AF[27]. Our intention was to identify the clinical signature of patients in the run up to an AF diagnosis and, in the absence of experimental data from a clinical trial, we performed analyses to identify this signature, working backwards from the time of AF. This approach is not new and is well established in the field of observational research[28]; it does constitute a novel aspect of our study when compared to prior predictive modelling exercises.

Considering a broader set of covariates could have improved further predictive performance over existing models; indeed, machine learning would be well suited to uncover new, previously unidentified risk factors from a large, unconstrained dataset. Data mining is not permissible when using the CPRD; alternative data sources, perhaps based on populations from outside the UK, could help to identify additional AF risk factors. Although we evaluated a number of readily implementable machine learning methods, consideration of more complex approaches (e.g., deep learning[29], XGBoost[30]) may yield an even greater improvement in predictive performance. We note that it was beyond the scope of this research to compare all potential machine learning models (at both baseline and when considering time-varying information). The choice for the selected models evaluated and reported in this study was informed by published clinical applications of machine learning techniques [31–34] and the experience of the study authors in selecting techniques suited to uncovering complex patterns of risk factor profiles from electronic healthcare records. Whilst the current study reports the relative performance of the final risk model and the next best preforming model evaluated, this was to illustrate the potential of machine learning techniques–and in particular our novel final risk score model—to uncover new patterns and associations that may inform the identification of patients at risk of or whom are undiagnosed with AF. Thus, the intention of the developed risk model in this study is to help inform future clinical and medical decision making; in this context, we demonstrate our model out-performs existing models, but recognise that other models may produce better or worse performance metrics. In the same vein, we considered machine learning techniques which were computationally feasible given the size of CPRD sample and the practical requirement for the final model to be implementable in routine clinical practice using available, tested and documented software packages.

A key limitation of this study is the CPRD, and therefore primary care records, as the single data source[35]. We likely underestimated AF incidence, as the condition is known to be underdiagnosed in the general population[6] and a high proportion of AF diagnoses are made incidentally in secondary care; although most secondary care diagnoses do appear in primary care, there may be a delay or some omissions[36]. While estimating AF incidence was not the aim of this study, it is unclear whether a single data source was sufficient to produce unbiased estimates of associations between risk factors and AF diagnoses. As may be expected, AF patient populations have been reported to differ between the CPRD and a secondary care source, Hospital Episodes Statistics (HES), given patients in secondary care may more frequently present with serious comorbidities[35].

CPRD does not provide suitable detailed information on ECG data and their coding—whether AF was a single episode or multiple episodes or persistent—this data is often left incomplete. In principle, sole use of the CPRD could introduce bias regarding the choice of AF predictors and the magnitude of their estimated effect sizes. However, similar associations between key cardiovascular risk factors and AF incidence across the CPRD, HES and the population-based Framingham Heart Study have been reported[36], implying a substantial bias may not apply to these covariates. A further limitation is that we were only able to analyse events chronologically in the order that they were recorded on the CPRD, which could impact the finding that a recent adverse cardiovascular event, such as heart failure, was a strong predictor of an upcoming AF diagnosis or that paroxysmal AF may have triggered the heart failure. It is possible that time lags exist in the recording of events, so that for some patients the AF diagnosis could have been made on the same day, or even prior to, the adverse cardiovascular event. Finally, our study is limited by its retrospective design, as we developed the model based on already diagnosed AF cases and without detailed information surrounding the diagnosis (for example, if patients presented with symptomatic AF or if the diagnosis was incidental) and real-world model performance would be likely to have reduced performance depending on the degree to which associations between risk factors and AF incidence are similar between ‘previously diagnosed’ and ‘yet undiagnosed’ AF cases.

The potential risks and benefits must be weighed against each other when considering implementation in clinical practice. If the model predicts a patient to be at risk of AF, that patient may be subjected to increased monitoring and either equipped with a novel device such as a watch, belt, patch or smartphone to provide ECG-based AF detection[25], or referred directly for a 12-lead ECG. Emerging technologies may make AF detection widely available and simple, at present they cannot be implemented e*n masse* in budget-constrained healthcare systems or systems where data volumes could overwhelm the personnel available to interpret the data, hence the need for improved methods to accurately identify patients at risk of AF. Additionally, the natural history of symptomless and undiagnosed AF compared with those with underlying comorbidities and symptomatic AF is unknown. Longitudinal studies to follow up on patients identified as potentially having undiagnosed AF would be required to determine the potential benefit to patients such as the use of anticoagulation therapy to reduce the risk of stroke in this patient group. The NN exhibited greater predictive performance than existing AF risk models and is a significant first step towards improving the detection of undiagnosed AF. We acknowledge that the real-world performance of our model is not certain, and it is unclear how well our findings from the time-varying NN will generalise to clinical practice. The next stage of the research is therefore to conduct a clinical trial to investigate model performance in practice, to assess how well the risk factors identified translate into predicting and diagnosing AF in a real-world setting in a bid to optimise the prevention of AF related stroke, heart failure and premature mortality.

## Supporting information

S1 Table Read codes used to define incident AF. (DOCX)

S2 Table Area under the receiver operator characteristic curve (AUROC) for risk models using baseline covariates. (DOCX)

S3 Table Time-varying logistic regression model output. (DOCX)

S4 Table Covariates considered in the baseline and time-varying neural networks. (DOCX)

S5 Table Optimisation of hyperparameters in candidate baseline and time-varying models. (DOCX)

S1 Fig Stylised illustration of study populations for risk models. A: baseline covariates; B: time-varying covariates.
(PPTX)

S2 Fig Study participation flow diagram. A total of 2,994,837 patients were aged ≥30 years without a five-year history of AF at the start of the study period, and had a complete set of key clinical measurements (height, weight, body mass index, diastolic blood pressure, systolic blood pressure) recorded over a one-year period. These patients were randomly assigned to training and holdout sets (2:1) to develop and assess the baseline neural network, respectively. The time-varying neural network was trained on all AF cases with at least one year of history at the time of AF and an age- and sex-matched sample of non-AF controls, and was evaluated after applying post-stratification weights to reflect the AF, age and sex composition of the full eligible population.
(TIF)

S3 Fig Predicted probabilities of AF for selected covariates in the time-varying neural network. (A) presence of heart failure in the latest quarter; (B) number of BMI, DBP and SBP measurements in the latest quarter; (C) maximum change in diastolic blood pressure and systolic blood pressure between the last two quarters; (D) most recent diastolic blood pressure and systolic blood pressure measurements.
Predicted probabilities were estimated by the method of partial dependence, whereby each predictor of interest was varied, and all other predictors were fixed at their patient-level observed values. Predicted probabilities were calculated on the unweighted training dataset for the time-varying neural network.
(TIF)
