# Supplementary material for: Predicting atrial fibrillation in primary care using machine learning
Source: PLoS One. 2019 Nov 1;14(11):e0224582. doi: 10.1371/journal.pone.0224582 (PMC6824570; doi:10.1371/journal.pone.0224582)
Supplement: S1 Table — (DOCX) [file pone.0224582.s001.docx]

S1 Table. Read codes used to define incident AF.

| **Description** | **Read code** |
| --- | --- |
| Atrial fibrillation and flutter | G573.00 |
| Atrial fibrillation | G573000 |
| Atrial flutter | G573100 |
| Paroxysmal atrial fibrillation | G573200 |
| Non-rheumatic atrial fibrillation | G573300 |
| Permanent atrial fibrillation | G573400 |
| Persistent atrial fibrillation | G573500 |
| Paroxysmal atrial flutter | G573600 |
| Atrial fibrillation and flutter NOS | G573z00 |
| ECG: paroxysmal atrial tachy. | 3274.00 |
| Atrial premature depolarization | G576300 |
| Implantation of intravenous atrial overdrive pacemaker | 7936900 |
| History of atrial flutter | 14AR.00 |
| Perc transluminal ablation of atrial wall for atrial flutter | 793M100 |
| ECG: atrial flutter | 3273.00 |
| Atrial fibrillation monitoring | 662S.00 |
| ECG: atrial fibrillation | 3272.00 |
| Paroxysmal atrial tachycardia | G570000 |
| Excepted from atrial fibrillation qual indic: Inform dissent | 9hF1.00 |
| Exception reporting: atrial fibrillation quality indicators | 9hF..00 |
| Implant intravenous pacemaker for atrial fibrillation | 7936A00 |
| Referral to atrial fibrillation clinic | 8HTy.00 |
| Atrial fibrillation care pathway | 8CMW200 |
| Perc translum ablat conduct sys heart for atrial flutter NEC | 793M300 |
