# Supplementary material for: Predicting atrial fibrillation in primary care using machine learning
Source: PLoS One. 2019 Nov 1;14(11):e0224582. doi: 10.1371/journal.pone.0224582 (PMC6824570; doi:10.1371/journal.pone.0224582)
Supplement: S2 Table — (DOCX) [file pone.0224582.s002.docx]

S2 Table. Area under the receiver operator characteristic curve (AUROC) for risk models using baseline covariates.

| **Model** | **Training set** | **Holdout set** |
| --- | --- | --- |
| Neural network | 0.823 | 0.818 |
| Random forest | 0.810 | 0.812 |
| Support vector machines | 0.812 | 0.811 |
| Logistic LASSO | 0.804 | 0.811 |
| Cox regression | 0.791 | 0.797 |
| CHARGE-AF | 0.787 | 0.796 |
| Framingham | 0.791 | 0.793 |
| ARIC | 0.723 | 0.727 |

ARIC: Atherosclerosis Risk in Communities; LASSO: least absolute shrinkage and selector operator
