# Supplementary material for: Predicting atrial fibrillation in primary care using machine learning
Source: PLoS One. 2019 Nov 1;14(11):e0224582. doi: 10.1371/journal.pone.0224582 (PMC6824570; doi:10.1371/journal.pone.0224582)
Supplement: S3 Table — (DOCX) [file pone.0224582.s003.docx]

S3 Table. Time-varying logistic regression model output.

| **Variable** | **Coefficient estimate** | **Standard error** | **P-value** |
| --- | --- | --- | --- |
| Intercept | -1.9987 | 0.0823 | <0.0001 |
| Current age (years) | 0.0018 | 0.0006 | 0.0032 |
| Most recently recorded BMI (kg/m^2^) | 0.0238 | 0.0012 | <0.0001 |
| Most recently recorded DBP (mmHg) | 0.0093 | 0.0006 | <0.0001 |
| Number of DBP measurements recorded in the past year | 0.1136 | 0.0027 | <0.0001 |
| History of antihypertensive medication usage | 0.1303 | 0.0135 | <0.0001 |
| History of heart failure | 2.2340 | 0.0613 | <0.0001 |
| History of myocardial infarction | 0.7155 | 0.0791 | <0.0001 |
| History of left ventricular hypertrophy | 1.4797 | 0.1895 | <0.0001 |
| History of coronary heart disease | 1.1865 | 0.0446 | <0.0001 |
| History of congenital heart disease | 2.5072 | 1.0378 | 0.0157 |

BMI: body mass index; DBP diastolic blood pressure
