# Supplementary material for: Predicting atrial fibrillation in primary care using machine learning
Source: PLoS One. 2019 Nov 1;14(11):e0224582. doi: 10.1371/journal.pone.0224582 (PMC6824570; doi:10.1371/journal.pone.0224582)
Supplement: S4 Table — (DOCX) [file pone.0224582.s004.docx]

S4 Table. Covariates considered in the baseline and time-varying neural networks.

| **Baseline neural network** | |
| --- | --- |
| **Variable** | **Description** |
| ***Patient Characteristics*** | |
| Age | Age in years |
| Sex | Male or female |
| Race | White, black, other, unknown |
| Smoking status | Baseline status (current, former, passive, unknown) |
| Weight | Baseline weight in kilograms |
| Height | Baseline height in metres |
| Body mass index | Baseline body mass index in kg/m^2^ |
| Diastolic blood pressure | Baseline diastolic blood pressure in mmHg |
| Systolic blood pressure | Baseline systolic blood pressure in mmHg |
| Hypertension (diagnosed) | Hypertension diagnosed in five years prior to index date |
| Hypertension (medication) | Hypertension medication prescribed in five years prior to index date |
| ***Comorbidities*** | |
| Heart failure | History of heart failure in five years prior to index date |
| Coronary heart disease | History of coronary heart disease in five years prior to index date |
| Congenital heart disease | History of congenital heart disease in five years prior to index date |
| Myocardial infarction | History of myocardial infarction in five years prior to index date |
| Left ventricular hypertrophy | History of left ventricular hypertrophy in five years prior to index date |
| Type 1 diabetes | History of type 1 diabetes in five years prior to index date |
| Type 2 diabetes | History of type 2 diabetes in five years prior to index date |
| **Time-varying neural network** | |
| **Variable** | **Description** |
| ***Patient Characteristics*** | |
| Age | Age in years at start of each 91-day quarter |
| Sex | Male or female |
| Race | Known white or other |
| Smoking status | Known current smoker or other |
| Height | Latest recorded value |
| Weight | A new set of predictors was derived using clinical measurements over the year prior to AF date (or equivalent for matched non-AF patients):   - latest value recorded in each quarter - difference between latest and earliest values recorded in total - difference between min and max values in each quarter - difference between min and max values across successive quarters - difference between min and max values recorded in total - number of measurements recorded in each quarter - number of measurements recorded in total |
| Body mass index |  |
| Diastolic blood pressure |  |
| Systolic blood pressure |  |
| ***Comorbidities*** | |
| Hypertension (diagnosed) | For each comorbidity, a new set of predictors was derived to indicate whether an event was observed in each quarter over the year prior to AF diagnosis (or equivalent for matched non-AF patients), or at any time prior to this |
| Hypertension (medication) |  |
| Heart failure |  |
| Coronary heart disease |  |
| Congenital heart disease |  |
| Myocardial infarction |  |
| Left ventricular hypertrophy |  |
| Type 1 diabetes |  |
| Type 2 diabetes |  |
