# Supplementary material for: Predicting atrial fibrillation in primary care using machine learning
Source: PLoS One. 2019 Nov 1;14(11):e0224582. doi: 10.1371/journal.pone.0224582 (PMC6824570; doi:10.1371/journal.pone.0224582)
Supplement: S5 Table — (DOCX) [file pone.0224582.s005.docx]

**S5 Table. Optimisation of hyperparameters in candidate baseline and time-varying models.**

| **Candidate baseline models** | | |
| --- | --- | --- |
| **Approach** | **R package and function** | **Hyperparameters** |
| Cox regression | Package: Survival^1,2^  Function: coxph | N/A |
| Logistic LASSO | Package: glmnet^3^  Function: cv.glmnet | Penalty term (lambda): 0.003804186 |
| Support vector machines | Package: Kernlab^4^  Function: ksvm | Kernel function: Gassian radial basis  Inverse kernel width (sigma): -1.309563 |
| Random forests | Package: randomForest^5^  Function: randomForest | Number of trees: 500  Number of variables sampled at each split: 8  Minimum size of terminal nodes: 25 |
| Neural networks | Package: nnet ^6^  Function: nnet | Size of hidden layer: 8  Weight decay term: 0.07 |
| **Candidate time-varying models** | | |
| **Approach** | **R package and function** | **Hyperparameters** |
| Logistic regression | Package: stats^7^  Function: glm | N/A |
| Neural networks | Package: mlr^8^  Function: makeTuneWrapper | Size of hidden layer: 5  Weight decay term: 0.01 |

LASSO: least absolute shrinkage and selection operator

**References**

1. Therneau T. A Package for Survival Analysis. 2015. Available from: <https://CRAN.R-project.org/package=survival>.

2. Therneau T, Grambsch PM. Modeling Survival Data: Extending the Cox Model. Springer, New York. 2000.

3. Friedman J, Hastie T, Tibshirani R. Regularization Paths for Generalized Linear Models via Coordinate Descent. Journal of Statistical Software. 2010;33(1):1-22.

4. Karatzoglou A, Smola A, Hornik K, et al. kernlab - An S4 Package for Kernel Methods in R. Journal of Statistical Software. 2004;11(9):1-20.

5. Liaw A, Wiener M. Classification and Regression by randomForest. R News. 2002;2(3):18-22.

6. Venables WN, Ripley BD. Modern Applied Statistics with S. Fourth Edition. ed. Springer, New York. 2002.

7. The R Core Team. R: A language and environment for statistical computing. 2017. Available from: <https://www.R-project.org/>.

8. Bischl B, Lang M, Kotthoff L, et al. mlr: Machine Learning in R. Journal of Machine Learning Research. 2016;17(170):1-5.
