# Supplementary figures and images for: Predicting atrial fibrillation in primary care using machine learning
Source: PLoS One. 2019 Nov 1;14(11):e0224582. doi: 10.1371/journal.pone.0224582 (PMC6824570; doi:10.1371/journal.pone.0224582)

## Slide 1
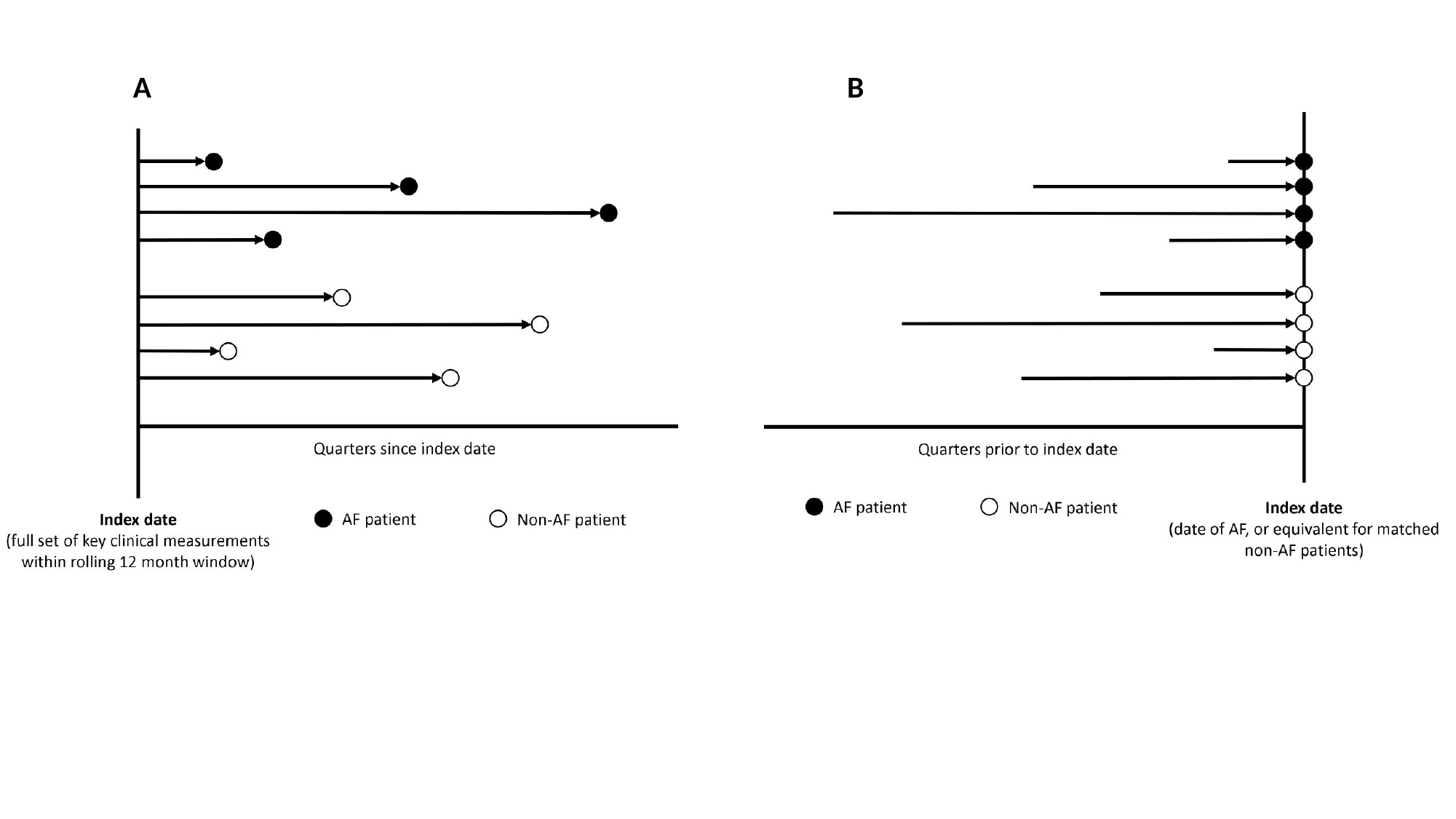

A
B

Supplement: S1 Fig — A: baseline covariates; B: time-varying covariates. (PPTX) [file pone.0224582.s006.pptx]

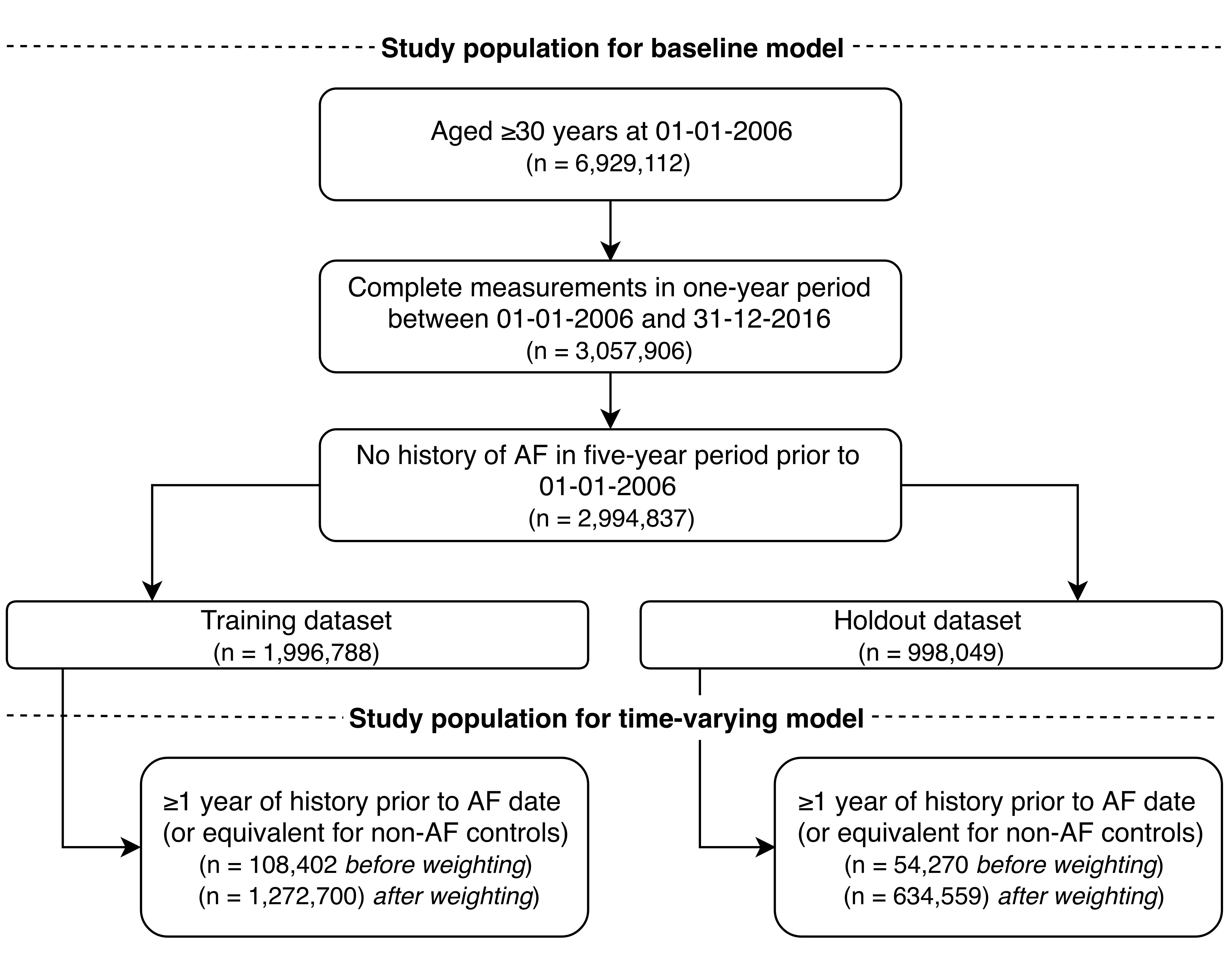

Supplement: S2 Fig — A total of 2,994,837 patients were aged ≥30 years without a five-year history of AF at the start of the study period, and had a complete set of key clinical measurements (height, weight, body mass index, diastolic blood pressure, systolic blood pressure) recorded over a one-year period. These patients were randomly assigned to training and holdout sets (2:1) to develop and assess the baseline neural network, respectively. The time-varying neural network was trained on all AF cases with at least one year of history at the time of AF and an age- and sex-matched sample of non-AF controls, and was evaluated after applying post-stratification weights to reflect the AF, age and sex composition of the full eligible population. (TIF) [file pone.0224582.s007.tif]

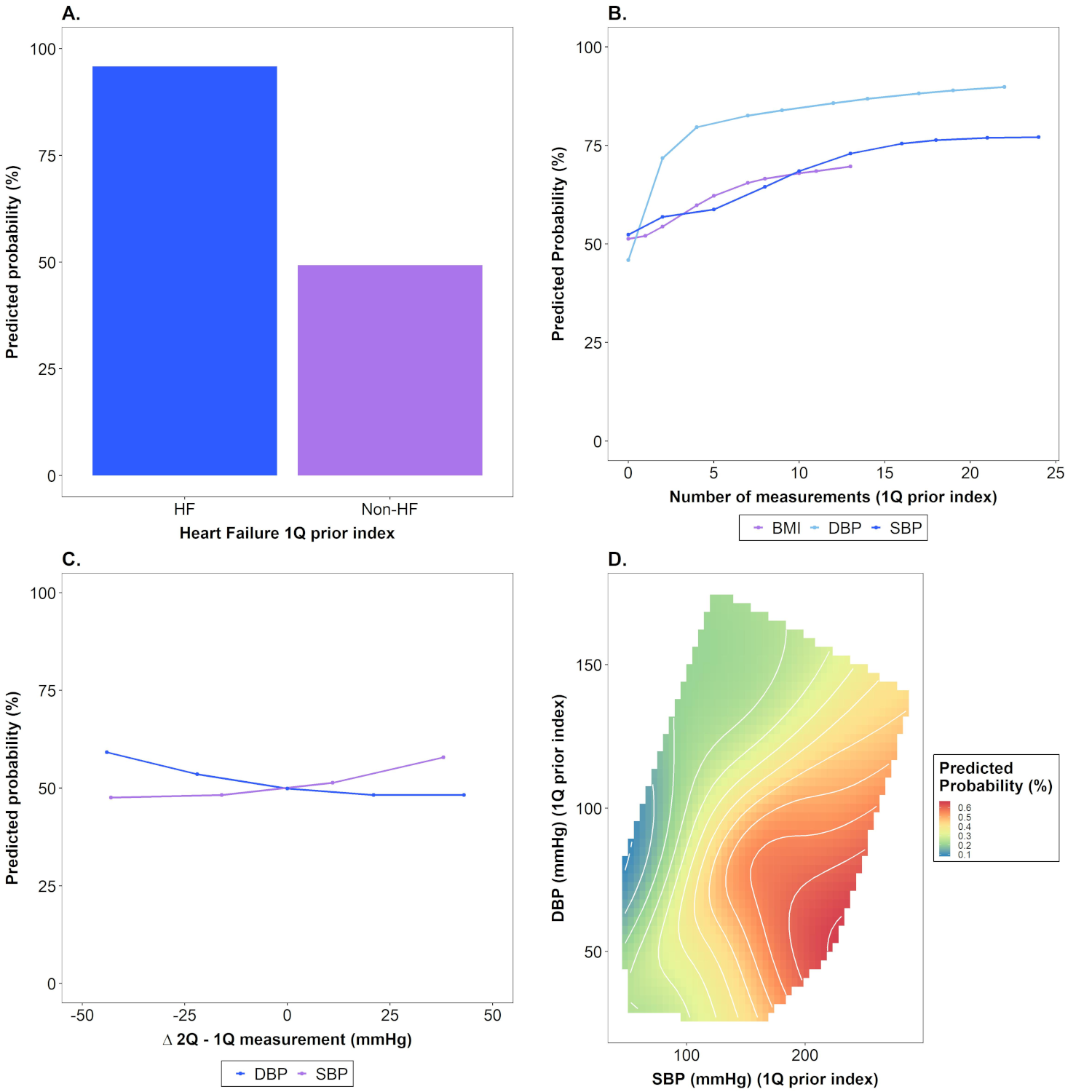

Supplement: S3 Fig — (A) presence of heart failure in the latest quarter; (B) number of BMI, DBP and SBP measurements in the latest quarter; (C) maximum change in diastolic blood pressure and systolic blood pressure between the last two quarters; (D) most recent diastolic blood pressure and systolic blood pressure measurements. Predicted probabilities were estimated by the method of partial dependence, whereby each predictor of interest was varied, and all other predictors were fixed at their patient-level observed values. Predicted probabilities were calculated on the unweighted training dataset for the time-varying neural network. (TIF) [file pone.0224582.s008.tif]
